# From Theory to Clinical Practice in the Use of GLP-1 Receptor Agonists and DPP-4 Inhibitors Therapy

**DOI:** 10.1155/2011/898913

**Published:** 2011-06-23

**Authors:** Ilaria Dicembrini, Laura Pala, Carlo Maria Rotella

**Affiliations:** Section of Endocrinology, Department of Clinical Pathophysiology, University of Florence, Viale Pieraccini 6, 50134 Florence, Italy

## Abstract

Promoting long-term adherence to lifestyle modification and choice of antidiabetic agent with low hypoglycemia risk profile and positive weight profile could be the most effective strategy in achieving sustained glycemic control and in reducing comorbidities. From this perspective, vast interest has been generated by glucagon-like peptide-1 (GLP-1) receptor agonists and dipeptidyl peptidase-4 inhibitors (DPP-4i). In this review our ten-year clinical and laboratory experience by *in vitro* and *in vivo* studies is reported. Herein, we reviewed available data on the efficacy and safety profile of GLP-1 receptor agonists and DPP-4i. The introduction of incretin hormone-based therapies represents a novel therapeutic strategy, because these drugs not only improve glycemia with minimal risk of hypoglycemia but also have other extraglycemic beneficial effects. In clinical studies, both GLP-1 receptor agonists and DPP-4i, improve **β** cell function indexes. All these agents showed trophic effects on beta-cell mass in animal studies. The use of these drugs is associated with positive or neucral effect on body weight and improvements in blood pressure, diabetic dyslipidemia, hepatic steazosis markets, and myocardial function. These effects have the potential to reduce the burden of cardiovascular disease, which is a major cause of mortality in patients with diabetes.

## 1. Introduction

The global prevalence of type 2 diabetic (T2DM) patients estimated at 6.4% is expected to be close to 8% by 2030 [1]. The overall total predicted increase is thought to be due largely to rising rates of overweight, obesity, physical inactivity, and population aging [2]. Improving glycaemic control remains the most effective therapeutic approach to reduce the risk of development and/or progression of microvascular complications. Furthermore, a recent meta-analysis of long-term, prospective randomized controlled clinical trials (UKPDS, PROactive, ADVANCE, VADT, and ACCORD) revealed a significant association between intensive blood glucose control and incident cardiovascular events: a 0.9% HbA1c decrease was related to a reduction of 17% in nonfatal MI (odds ratio (OR): 0.83, 95% confidence interval (CI): 0.75–0.93) and 15% in coronary heart disease (OR: 0.85, 95% CI: 0.77–0.93) versus conventional therapy [3]. In a metaregression analysis, higher body mass index (BMI), duration of diabetes, and incidence of severe hypoglycaemia were associated with greater risk of cardiovascular death in intensive treatment groups [4]. Altogether, these results underline the importance of achieving and maintaining good glycemic control, from the time of diagnosis, predominantly through a tailored approach. Promoting long-term adherence to lifestyle modification and choice of antidiabetic agent with low hypoglycemia risk profile and positive weight profile could be the most effective strategy in achieving sustained glycemic control and in reducing comorbidities. From this perspective, vast interest has been generated by glucagon-like peptide-1 (GLP-1) receptor agonists and dipeptidyl peptidase-4 inhibitors (DPP-4i) based on many clinical studies revealing long-term glucose-lowering efficacy related to low hypoglycemic rates, positive/neutral weight effects, and amelioration of *β* cell function [5–7].

## 2. Background: A Ten-Year Clinical and Laboratory Experience

GLP-1 is a gastrointestinal hormone, mainly secreted in a nutrient-dependent manner, which enhances glucose-induced insulin secretion and induces satiety. It has been reported that GLP-1 levels after an oral glucose load are reduced in patients with T2DM [8] even if more recent data suggest a controversial point of view [9]. The reduction of oral glucose-stimulated active GLP-1 levels in T2DM patients has also been observed during euglycaemic hyperinsulinemic clamp. This impairment, which is not the result of differences in glycaemia or insulinaemia during assessment, could contribute to the pathogenesis of hyperglycaemia in T2DM [8] and in particular to the reduction of early postprandial insulin secretion; in fact, the administration of GLP-1 receptor antagonists to healthy volunteers elicits both an impairment of meal-induced insulin secretion and an increase of postprandial glycaemia similar to that observed in T2DM. GLP-1 is rapidly inactivated by dipeptidyl peptidase-4 (DPP-4), an enzyme produced by endothelial cells in different districts and that circulates in plasma. The reduction of meal or oral-glucose-stimulated GLP-1 levels in T2DM patients is probably due to both an impairment of secretion and an increased degradation. The major limitation of using native GLP-1 to treat diabetic patients is the short half-life. There are now several compounds in various stages of preclinical or clinical development for the treatment of T2DM that utilize the GLP-1 signaling pathway; these include GLP-1 receptor agonists with extended half-lives and DPP-4i that increase circulating levels of endogenous, intact, and bioactive GLP-1 [10].

Metformin, first drug of choice in the treatment of T2DM, induced a significant increase of GLP-1(7–36) amide/(7–37) at 30 and 60 min after the oral glucose load in obese nondiabetic subjects. In pooled human plasma, metformin (0.1–0.5 microg/mL) significantly inhibited degradation of GLP-1(7–36) amide after a 30 min incubation at 37°C; similar results were obtained in a buffer solution containing DPP-4. This effect could be due to an inhibition of GLP-1 degradation [11]. This effect was also present in obese drug-naïve T2DM patients. In fact 4 weeks after treatment with metformin 850 mg three times daily, post-load GLP-1 levels was significantly increased [12]. Furthermore the relationship between meal-induced GLP-1 secretion and postprandial hyperglycemia was studied in 21 drug-naïve T2DM patients. Blood glucose and active GLP-1 levels were measured 0, 30, 60, 90, and 120 min after a standard meal test, and a continuous glucose monitoring (CGM) system was applied for the following 3 days. A significant inverse correlation between GLP-1 response and postprandial glucose levels was observed for each additional unit of total energy or carbohydrate intake. A lower GLP-1 response is associated with higher levels of HbA1c and with a greater degree of meal-induced hyperglycemia, both during a meal test and “real-life” condition [13].

GLP-1 and DPP-4 activity levels have been subsequently studied during an oral glucose tolerance test (OGTT), in order to investigate their modification in patients with different glucose tolerance degree. At 30 min GLP-1 levels were significantly lower in subjects with impaired glucose tolerance and T2DM compared to those with normal glucose tolerance. The area under the curve of GLP-1 levels during OGTT was significantly different among the three groups with a significant decrease between subjects with normal and impaired glucose tolerance and between those with normal glucose tolerance and type 2 diabetes mellitus. DPP-4 showed no significant difference between the groups. Therefore, an increase of GLP-1 degradation seems to be not involved in the early stages of diabetes [14]. However, chronic hyperglycaemia was significantly associated to an increased DPP-4 activity and mRNA expression in human glomerular endothelial cells in vitro [15]. In order to assess effects of chronic hyperglycaemia on circulating DPP-IV activity in vivo, we have analyzed diabetic patients not adequately controlled. T2DM subjects with HbA1c levels >8.5% showed significantly higher DPP-4 activity than patients affected by newly diagnosed diabetes and Impaired Glucose Tolerance (IGT). Variations in DPP-4 activity over 3 months in T2DM showed a significant positive correlation with HbA1C levels. Therefore, chronic hyperglycaemia induced a significant increase in DPP-4 activity in type 2 diabetic patients with poor metabolic control, probably contributing to the reduction in circulating active GLP-1 and subsequently to postprandial hyperglycaemia [16]. Moreover, the effects of insulin sensitizers drugs, such as Metformin and Rosiglitazone, on the modulation of GLP-1 circulating levels, DPP-4 activity, and mRNA expression was evaluated in human aortic endothelial cells (HAECs) and human microvascular dermal endothelial cells (HMVECs) exposed to high glucose concentration. Hyperglycemia is associated to a significant DPP-4 activity increase only in microvascular endothelial cells. Rosiglitazone is able to modulate in a negative manner the expression of DPP-4 but not its activity in macrovascular endothelial cells, while at 24 h of exposure it is able to increase significantly DPP-4 activity but not its expression in microvascular endothelial cells. Metformin at 48 h in microvascular endothelial cells is able to reduce in a significant manner the activity of DPP-4 but not its expression [17]. Therefore, the modulation of DPP-4 seems to be site specific.

## 3. Rationale and Extraglycaemic Effects of Incretin Therapy

GLP-1 receptor agonists that resist degradation by DPP-4 and have protracted-action kinetics have been developed, and DPP-4i that slow the enzymatic cleavage of native GLP-1 provide alternative approaches to enhancing incretin-mediated glucose control. 

However, GLP-1 receptor agonists and DPP-4i are premised on highly divergent mechanisms of action. DPP-4 is ubiquitously expressed in many tissues and is involved in a wide range of processes in addition to its influence on incretin hormone biological effects. GLP-1 receptor agonists provide pharmacologic levels of GLP-1 receptor stimulation, whereas DPP-4i appear to increase circulating levels of GLP-1 to within the physiologic range [18]. 

GLP-1 receptors are additionally expressed in extrapancreatic tissue, having potential for the treatment of obesity and for beneficial cardio- and endothelioprotective effects. Because diabetic patients are prone to cardiovascular disease, T2DM treatment strategies should address the cardiovascular risk profile, including blood pressure, lipids, and body weight profile, in addition to intensive glycaemic control. 

Incretin-based therapies, both GLP-1 receptor agonists and DPP-4i, unlike many other antidiabetic therapies (such as sulphonylureas and insulin in particular), they do not induce weight gain. Moreover therapy with GLP-1 receptor agonists results in progressive and sustained significant weight loss in most patients [19, 20]. These agents exert physiological effect similar to those of native GLP-1, including enhancement of glucose-dependent insulin secretion and suppression of inappropriately high glucagon secretion. Furthermore, they also slow gastric emptying and reduce food intake. DPP-4i have similar actions to that of GLP-1 receptor agonists; they also enhance glucose-dependent insulin secretion and suppress glucagon, but they do not delay gastric emptying or reduce food intake. In controlled clinical trials the effect of DPP4i on weight was neutral [21]. 

In a recent large retrospective study exenatide twice daily was compared with other glucose-lowering agents in terms of their impact on cardiovascular events incidence. Despite the higher prevalence of coronary artery disease (CAD), obesity, hyperlipidemia, hypertension, and/or other comorbidities at baseline, exenatide-treated patients were less likely to have a CVD event than non-exenatide-treated ones (HR: 0.81, 95% CI: 0.68–0.95; *P* = .01). Furthermore, exenatide-treated patients demonstrated lower rates of CVD-related hospitalization (HR: 0.88, 95% CI: 0.79–0.98; *P* = .02) and all-cause hospitalization (HR: 0.94, 95% CI: 0.91–0.97; *P* < .001) than those not having received exenatide [22].

Emerging data suggest a cardioprotective effect of DPP-4i in humans. These drugs reduce HbA1c, although to a lesser extent than sulphonylureas, with no weight gain and low hypoglycaemic risk. The risk of cardiovascular events and all-cause death, during DPP-4i treatment, is 0.76 [0.46–1.28] and 0.78 [0.40–1.51], respectively [23]. In particular, sitagliptin administration at a single dose of 100 mg in patients with CAD and preserved left ventricular (LV) function enhanced LV response to stress, attenuated postischaemic stunning, and improved global and regional LV performance compared to placebo [24]. Encouraging results have also been published from a phase III randomized placebo-controlled trial regarding the granulocyte-colony stimulating factor- (G-CSF) based stem cell mobilization in combination with sitagliptin in patients after acute MI. During the first 6 weeks of followup, sitagliptin along with G-CSF seems to be safe and effective for myocardial regeneration representing a new therapeutic option in the future [25].

There are also data regarding the impact of GLP-1 receptor agonists and DPP-4i on endothelial function and cardiovascular diseases biomarkers. In particular, an additional effect of liraglutide on inflammatory process has been reported, since it was related to a significant reduction of high-sensitivity C-reactive protein (hsCRP) circulating levels in T2DM patients through a dose-dependent pathway [26]. Similar inhibitory effects on vascular cell adhesion molecule-1 (VCAM-1) and hsCRP have been reported for exenatide [27, 28]. Protective effects on endothelial function have been also described for sitagliptin, mainly through induction of NOS activity, and to greater extent in comparison to pioglitazone [29]. 

Substantial evidence demonstrates that both GLP-1 receptor agonists and DPP-4i produce modest reductions in systolic blood pressure and, in some cases, diastolic blood pressure. The effect of the incretin hormones on serum lipids are either neutral or beneficial, with small, nonsignificant decreases in low-density lipoprotein cholesterol, increases in high-density lipoprotein cholesterol, and occasionally significant decreases in fasting triglyceride levels. Furthermore, GLP-1 receptor agonists showed positive effects on hepatic steatosis [30, 31]. 

T2DM is a progressive chronic disease characterized by insulin resistance and impaired beta-cell function. Treatments that prevent further beta-cell decline are therefore essential for the management of type 2 diabetes. In vitro and animal studies showed that GLP-1 promotes *β*-cell neogenesis and preservation and inhibits *β*-cell apoptosis [32]. Various human studies confirmed GLP-1 receptor agonists and DPP-4i effects in *β*-cell function improvement [33–35] but there is no evidence supporting *β*-cell proliferation and/or antiapoptosis effect in human yet. 

Table 1 summarizes the differences between GLP-1 receptors agonists and DPP-4 inhibitors.

## 4. Clinical Use of Glucagon-Like Peptide 1 Receptor Agonists and Dipeptidyl Peptidase 4 Inhibitors

### 4.1. GLP-1 Receptor Agonists

GLP-1 receptor agonists have, to date, varying degrees of evidence to support their positive effects on glycated hemoglobin (HbA1c) reduction in T2DM patients.

Efficacy and safety data for exenatide were derived from three phase III Diabetes Management for Improving Glucose Outcomes (AMIGO) trials in patients with T2DM who were inadequately controlled with either a sulfonylurea, metformin, or a sulfonylurea plus metformin [36–38].

In each of these trials, twice daily exenatide was shown to reduce HbA1c significantly, by approximately 0.9% from baseline over the 30-week trial period compared with placebo. A pooled analysis of trial data plus two 52-week extension studies, which were completed by 314 of 1446 patients in an intention-to-treat group from the 30-week randomized trials, found that the reduction in HbA1c was sustained over 2 years [39].

In T2DM patients not adequately controlled with metformin, exenatide showed noninferiority effects on HbA1c in comparison to basal insulin glargine add-on therapy, but a better beta-cell function improvement and weight profile (difference −4.6 kg, *P* < .0001) [40]. 

In two open-label trials, exenatide was compared with insulin treatment in patients inadequately controlled by metformin plus sulphonylurea. In the first study, 551 patients were randomized to receive glargine once daily or exenatide twice daily. After 26 weeks, HbA1c fell by 1.1% points in both groups (baseline: 8.2%). Exenatide reduced postprandial glucose excursions more than insulin glargine, while insulin glargine had a more pronounced effect on fasting plasma glucose. Body weight increased, as expected, by 1.8 kg with glargine while a 2.3 kg weight loss was obtained with exenatide. Nocturnal hypoglycaemia was less frequent with exenatide [41]. Barnett et al. confirmed these differences in T2DM not adequately controlled with metformin or sulphonylurea [42]. In the second 52-week trial, exenatide was compared with twice daily biphasic insulin aspart. Exenatide treatment resulted in HbA1c reduction similar to biphasic insulin aspart and provided better postprandial glycaemic control and weight profile (−2.5 kg versus +2.9 kg) [43]. 

A recent meta-analysis of placebo-controlled clinical trials assessing efficacy of incretin-based medications in T2DM patients showed that liraglutide both at 1.2 and 1.8 mg is associated with greater reductions in HbA1c in comparison to exenatide (weighted mean differences, WMD = −0.75, 95% CI = −0.83 to −0.67, *P* < .001), vildagliptin (WMD = −0.67, 95% CI = −0.83 to −0.52, *P* < .001), or sitagliptin (WMD = −0.79, 95% CI = −0.93 to −0.65, *P* < .001) [44]. In T2DM not adequately controlled with metformin, exenatide and sulphonylurea are associated to similar improvement in HbA1c levels, fasting and post prandial glycemia. However exenatide add-on therapy induced weight loss, improvement of insulin resistance indexes, and high-sensitivity C-reactive protein (Hs-CRP) reduction versus glibenclamide [45]. 

Liraglutide's efficacy and safety in combination with other hypoglycemic treatment has been extensively investigated in the phase III Liraglutide Effect and Action in Diabetes (LEAD) clinical development programme. Trials duration ranged from 26 to 52 weeks, and several trials have ongoing extension phases. In T2DM patients not adequately controlled with sulphonylurea, liraglutide add-on therapy resulted, in comparison to rosiglitazone, in significantly greater HbA1c reduction (treatment differences for liraglutide 1.8 mg −0.7% (95% CI 1.6; 1.1) in comparison to rosiglitazone, and −0.6% (95% CI 1.5; 1.1) for liraglutide 1.2 mg), and in a significantly better weight profile [46]. Liraglutide in addition to metformin showed a noninferior glycemic amelioration in comparison to sulphonylurea, associated to a relatively low rate of reported minor hypoglycemia (0.03–0.14 events/year) significantly less than for the glimepiride group (1.23 events/year;  *P* = .001) [47]. In T2DM patients not adequately controlled with metformin and sulphonylurea, liraglutide addition was significantly associated to greater reduction in HbA1c in comparison to insulin glargine (treatment differences for liraglutide versus insulin glargine −0.24%, 95% CI −0.39, −0.08; *P* = .0015), positive weight effects (mean treatment difference of −3.43 kg (95% CI −4.00, −2.86; *P* < .0001) and waist circumference reductions (treatment difference −2.40 cm, 95% CI −3.14, −1.65; *P* < .0001) [48]. 

LEAD-6 study (26 week) confirmed, during liraglutide (1.8 mg) add-on therapy, a significantly greater reduction of HbA1c and fasting glycemia, less effective postprandial glucose control, and similar weight effects (liraglutide −3.24 kg versus exenatide −2.87 kg) in comparison to exenatide (10 *μ*g) [49].

Currently, concomitant use of GLP-1 receptor agonist and insulin is not recommended by the manufacturer. Potential concerns with combination therapy include additive hypoglycemia, reduced patient adherence to another subcutaneously administered medication, and an estimated $200–300 additional monthly cost for the patient. In a retrospective analysis evaluating the effectiveness of exenatide and insulin combination (*N* = 76) for 1 year, statistically significant reductions in HbA1c (mean 0.87% reduction) and weight change (mean 5.2 kg reduction) were observed. As reported in the other clinical trials, there were significant reductions especially in bolus insulin dose, with 45% of patients discontinuing short-acting insulin and a 35% reduction in the mean daily dose of short-acting insulin [50]. These compounds were safe; in fact the most common side effects are nausea, vomiting and diarrhea reported especially at the beginning of the therapy; side effects as pancreatitis and QT prolongation during exenatide treatment are rare [18].

### 4.2. Dpp-4 Inhibitors

DPP-4i, also called incretin enhancers, may be used as monotherapy or in combination with other antidiabetic compounds. Sitagliptin, vildagliptin, and saxagliptin are already available in many countries, either as single agents or in fixed-dose combined formulations with metformin. Other DPP-4i, such as alogliptin and linagliptin, are currently in late phase of development. 

All together gliptins have a good oral bioavailability which is not significantly influenced by food intake. Pharmacokinetic/dynamic characteristics, as sufficiently prolonged half-life and sustained DPP-4 enzyme inactivation, generally allow one single oral administration per day for the management of T2DM; the only exception is vildagliptin for which a twice daily administration is recommended because of a shorter half-life. DPP-4i are in general not substrates for cytochrome P450 (except saxagliptin that is metabolized via CYP 3A4/A5) and do not act as inducers or inhibitors of this system [51, 52]. Several metabolites have been documented but most of them are inactive; however, the main metabolite of saxagliptin also exerts a significant DPP-4 inhibition and is half as potent as the parent compound. Renal excretion is the most important elimination pathway, except for linagliptin whose excretion in the liver appears to be predominant. PK properties of gliptins, combined with their good safety profile, explain why no dose adjustment is necessary in elderly patients or in patients with mild to moderate hepatic impairment. As far as patients with renal impairment are concerned, significant increases in drug exposure for sitagliptin and saxagliptin have been reported so that appropriate reductions in daily dosages are recommended according to estimated glomerular filtration rate. The PK characteristics of DPP-4i suggest that these compounds are not exposed to a high risk of drug-drug interactions [53]. However, the daily dose of saxagliptin should be reduced when coadministered with potent CYP 3A4 inhibitors. The DPP-4i improve glycaemic control, reducing both fasting and postprandial glucose levels to lower HbA1c levels, without weight gain and with an apparently good adverse event profile. At present, there seems to be little to distinguish between the different inhibitors in terms of their efficacy as antidiabetic agents and their safety. In particular, vildagliptin monotherapy resulted in improved glycemic control in drug-naïve patients with type 2 diabetes [54]. Although the hypothesis of noninferiority to gliclazide was not borne out statistically, the reductions in HbA1c were similar over a two-year period and vildagliptin had significant benefits in terms of less weight gain and less hypoglycemia. Vildagliptin improves islet function in T2DM under fasting conditions [55]. This suggests that DPP-4 inhibition has metabolic benefits in addition to enhancing meal-induced GLP-1 and GIP activity. The weight neutrality of vildagliptin likely results in part from its intrinsically low risk for hypoglycemia [56]. Recent studies point to additional potential mechanisms. One study found that drug-naïve patients randomized to vildagliptin exhibited significantly lower chylomicron lipid and apolipoprotein levels than placebo patients, suggesting that vildagliptin may inhibit intestinal fat extraction. A meta-analysis indicates that vildagliptin was not associated with increased risk of hepatic events or hepatic enzyme elevations indicative of drug-induced liver injury, pancreatitis, infections, or skin-related toxicity [57]. The safety of vildagliptin in renal transplant patients has been recently assessed [58, 59]. 

Sitagliptin monotherapy improved glycemic control compared to placebo and was generally well tolerated in patients with type 2 diabetes. The glycemic response to treatment with sitagliptin 100 mg/day was similar between 100-mg once daily and 50-mg twice daily dose regimens [60]. Recent trials have shown that the combinations offer additive efficacy in reducing blood glucose when given as initial antidiabetic therapy and as add-on therapy when pioglitazone alone fails to maintain glycemic control. The combination of sitagliptin and pioglitazone was well tolerated and would appear to be suited to a fixed-dose single-tablet combination for once daily administration [61].

Preclinical and clinical trial data with sitagliptin to date do not indicate an increased risk of pancreatitis [62]. 

Saxagliptin, 5 mg once daily, has been shown to be effective in patients with type 2 diabetes treated with diet alone, metformin, sulfonylurea, or glitazone, with a favourable tolerance profile. Reduction in HbA1c levels averaged 0.6−0.8%, without increasing the risk of hypoglycaemia or promoting weight gain [63]. In clinical trials, saxagliptin as monotherapy or in combination with metformin, a sulphonylurea (glyburide), or thiazolidinedione (pioglitazone or rosiglitazone) significantly improved glycemic control without increasing hypoglycaemic events and weight versus comparator [64–68]. Furthermore, recent clinical data supported the efficacy and favorable tolerability profile of a reduced daily dose of saxagliptin (2.5 mg) in T2DM patients with a significant renal impairment [69]. In conclusion, DPP-4i have emerged as alternatives to sulfonylureas, commonly used as add-on to metformin in treatment of T2DM. They showed similar efficacy to sulfonylureas but with lower risk of hypoglycemia and reduction or no change in body weight, and if confirmed in humans, they may preserve islet function thereby minimizing the risk for secondary failure. Their limitation at present is the lack of long-term experience on durability and safety [21].

## 5. Future Perspective

Few head-to-head clinical trials comparing different incretin-based drugs as add-on treatment are performed. Scheen et al. showed similar efficacy on glycaemia and tolerability between saxagliptin and sitagliptin added to metformin in T2DM patients inadequately controlled [70]. Liraglutide provided significant better improvement in glycemic control, especially in fasting condition, in comparison to exenatide twice daily [49]. Exenatide once weekly was associated with significant greater HbA1c reduction (−1.5%, 95% CI −1.7 to −1.4 versus −0.9%, −1.1 to −0.7) and weight loss (−2.3 kg, 95% CI −2.9 to −1.7 versus −1.5 kg, 95% CI −2.4 to −0.7) in comparison to sitagliptin as add-on treatment to metformin [71].

The profile of action of GLP-1 receptor agonists and DPP-4 inhibitors suggests the possibility of an actual reduction in cardiovascular risk, which needs to be confirmed by large long-term clinical trials, and beta cell protection, to date only proven in animal models [72]. The use of incretins is suggested very early in the type 2 diabetic history, but some studies suggest a potential use of them also in obese patients and in prediabetic patients [73, 74]. Moreover, recently liraglutide efficacy was evaluated in the management of obese patients. This treatment was well tolerated during 20 weeks and associated to a significant higher weight loss in comparison to placebo and orlistat [73]. 

At present, GLP-1 receptor agonists need to be administered by subcutaneous injection once or twice daily. Several once weekly GLP-1 receptor agonists are in phase 3 clinical trial development as exenatide once weekly, taspoglutide, albiglutide, LY2189265, and CJC-1134-PC. A greater reduction in HbA1c and fasting plasma glucose was found with the once weekly GLP-1 receptor agonists compared with exenatide BID, while the effect on postprandial hyperglycemia was modest. The reduction in HbA1c was in most studies greater compared to oral antidiabetic drugs and insulin glargine. The reduction in weight did not differ between the short- and long-acting agonists. The gastrointestinal side effects were less with the once weekly agonists compared with exenatide BID, except for taspoglutide. Antibodies seem to be most frequent with exenatide once weekly, while hypersensitivity has been described in few patients treated with taspoglutide. The development of taspoglutide has been stopped because of safety problems. Injection site reactions differ among the long-acting GLP-1 receptor agonists and are observed more frequently than with exenatide BID and liraglutide. The once weekly GLP-1 receptor agonists are promising candidates for the treatment of type 2 diabetes although their efficacy may not be superior to once daily analogue liraglutide [75]*.*

Hyperglycemia plays a major role in the development of the microvascular and macrovascular complications of diabetes. With the exception of metformin, traditional agents used for the treatment of type 2 diabetes are able to improve glycemia, but their use is often limited by treatment-associated side effects, including hypoglycemia, weight gain, and edema and they do not have any positive effect on beta-cell mass or function. The introduction of incretin hormone-based therapies represents a novel therapeutic strategy, because these drugs not only improve glycemia with minimal risk of hypoglycemia but also have other extraglycemic beneficial effects. In clinical studies, both exenatide and liraglutide, such as sitagliptin, vildagliptin, and saxagliptin, improve *β* cell function and glycemia with minimal hypoglycemia. All these agents have trophic effects on beta-cell mass in animal studies. The use of these agents is also associated with reduced or neutral effect on body weight and improvements in blood pressure, diabetic dyslipidemia, hepatic steatosis markers, and myocardial function. These effects have the potential to reduce the burden of cardiovascular disease, which is a major cause of mortality in patients with diabetes.

## Figures and Tables

**Table 1:** 

	GLP-1 receptor agonists	DPP-4 inhibitors
Administration	Injection	Oral
GLP-1 concentration	Pharmacologic	Physiologic
Insulin Secretion	++	+
Glucagon secretion	− −	− −
Action	↑ GLP-1	↑ GLP-1 and GIP
Gastric emptying	−	±
Weight loss	++	=
*β*-cell protection	++*	++*
Immunogenicity	+°	−
Nausea and vomiting	+	−

*In animal model.

°Not similar for different GLP-1 receptor agonists according to their homology to native peptide.

## References

[1] Shaw JE, Sicree RA, Zimmet PZ (2010). Global estimates of the prevalence of diabetes for 2010 and 2030. *Diabetes Research and Clinical Practice*.

[2] Sturm R (2008). Stemming the global obesity epidemic: what can we learn from data about social and economic trends?. *Public Health*.

[3] Ray KK, Seshasai SRK, Wijesuriya S (2009). Effect of intensive control of glucose on cardiovascular outcomes and death in patients with diabetes mellitus: a meta-analysis of randomised controlled trials. *Lancet*.

[4] Mannucci E, Monami M, Lamanna C (2009). Prevention of cardiovascular disease through glycemic control in type 2 diabetes: a meta-analysis of randomized clinical trials. *Nutrition, Metabolism and Cardiovascular Diseases*.

[5] Preumont V, Hermans MP, Brichard S, Buysschaert M (2010). Six-month exenatide improves HOMA hyperbolic product in type 2 diabetic patients mostly by enhancing beta-cell function rather than insulin sensitivity. *Diabetes Metab*.

[6] Karasik A, Aschner P, Katzeff H, Davies MJ, Stein PP (2008). Sitagliptin, a DPP-4 inhibitor for the treatment of patients with type 2 diabetes: a review of recent clinical trials. *Current Medical Research and Opinion*.

[7] Ahrén B, Pacini G, Tura A, Foley JE, Schweizer A (2007). Improved meal-related insulin processing contributes to the enhancement of B-cell function by the DPP-4 inhibitor vildagliptin in patients with type 2 diabetes. *Hormone and Metabolic Research*.

[8] Mannucci E, Ognibene A, Cremasco F (2000). Glucagon-like peptide (GLP)-1 and leptin concentrations in obese patients with type 2 diabetes mellitus. *Diabetic Medicine*.

[9] Nauck MA, Vardarli I, Deacon CF, Holst JJ, Meier JJ Secretion of glucagon-like peptide-1 (GLP-1) in type 2 diabetes: what is up, what is down?. *Diabetologia*.

[10] Rotella CM, Pala L, Mannucci E (2005). Glucagon-like peptide 1 (GLP-1) and metabolic diseases. *Journal of Endocrinological Investigation*.

[11] Mannucci E, Ognibene A, Cremasco F (2001). Effect of metformin on glucagon-like peptide 1 (GLP-1) and leptin levels in obese nondiabetic subjects. *Diabetes Care*.

[12] Mannucci E, Tesi F, Bardini G (2004). Effects of metformin on glucagon-like peptide-1 levels in obese patients with and without type 2 diabetes. *Diabetes, nutrition &amp; metabolism*.

[13] Mannucci E, Pala L, Monami M (2010). Glucagon-like peptide-1 response to meals and post-prandial hyperglycemia in type 2 diabetic patients. *Journal of Endocrinological Investigation*.

[14] Pala L, Ciani S, Dicembrini I (2010). Relationship between GLP-1 levels and dipeptidyl peptidase-4 activity in different glucose tolerance conditions. *Diabetic Medicine*.

[15] Pala L, Mannucci E, Pezzatini A (2003). Dipeptidyl peptidase-IV expression and activity in human glomerular endothelial cells. *Biochemical and Biophysical Research Communications*.

[16] Mannucci E, Pala L, Ciani S (2005). Hyperglycaemia increases dipeptidyl peptidase IV activity in diabetes mellitus. *Diabetologia*.

[17] Pala L, Pezzatini A, Dicembrini I Different modulation of dipeptidyl peptidase-4 activity between microvascular and macrovascular human endothelial cells.

[18] Nauck MA (2011). Incretin-based therapies for type 2 diabetes mellitus: properties, functions, and clinical implications. *American Journal of Medicine*.

[19] Varanasi A, Chaudhuri A, Dhindsa S (2010). Durability of effects of exenatide treatment on glycemic control, body weight, systolic blood pressure, Crp and triglyceride concentrations. *Endocrine Practice*.

[20] Davies MJ, Kela R, Khunti K (2011). Overview of the preclinical and clinical data and its role in the treatment of type 2 diabetes. *Diabetes, Obesity and Metabolism*.

[21] Ahrén B Are sulfonylureas less desirable than DPP-4 inhibitors as add-on to metformin in the treatment of type 2 diabetes?.

[22] Best JH, Hoogwerf BJ, Herman WH (2011). Risk of cardiovascular disease events in patients with type 2 diabetes prescribed the glucagon-like peptide 1 (GLP-1) receptor agonist exenatide twice daily or other glucose-lowering therapies: a retrospective analysis of the lifeLink database. *Diabetes Care*.

[23] Monami M, Iacomelli I, Marchionni N, Mannucci E (2010). Dipeptydil peptidase-4 inhibitors in type 2 diabetes: a meta-analysis of randomized clinical trials. *Nutrition, Metabolism and Cardiovascular Diseases*.

[24] Read PA, Khan FZ, Heck PM, Hoole SP, Dutka DP (2010). DPP-4 inhibition by sitagliptin improves the myocardial response to dobutamine stress and mitigates stunning in a pilot study of patients with coronary artery disease. *Circulation: Cardiovascular Imaging*.

[25] Theiss HD, Brenner C, Engelmann MG (2010). Safety and efficacy of SITAgliptin plus GRanulocyte-colony-stimulating factor in patients suffering from acute myocardial infarction (SITAGRAMI-Trial)–Rationale, design and first interim analysis. *International Journal of Cardiology*.

[26] Courrèges JP, Vilsbøll T, Zdravkovic M (2008). Beneficial effects of once-daily liraglutide, a human glucagon-like peptide-1 analogue, on cardiovascular risk biomarkers in patients with type 2 diabetes. *Diabetic Medicine*.

[27] Viswanathan P, Chaudhuri A, Bhatia R, Al-Atrash F, Mohanty P, Dandona P (2007). Exenatide therapy in obese patients with type 2 diabetes mellitus treated with insulin. *Endocrine Practice*.

[28] Arakawa M, Mita T, Azuma K (2010). Inhibition of monocyte adhesion to endothelial cells and attenuation of atherosclerotic lesion by a glucagon-like peptide-1 receptor agonist, exendin-4. *Diabetes*.

[29] Ye Y, Keyes KT, Zhang C, Perez-Polo JR, Lin YU, Birnbaum Y (2010). The myocardial infarct size-limiting effect of sitagliptin is PKA-dependent, whereas the protective effect of pioglitazone is partially dependent on PKA. *American Journal of Physiology*.

[30] Klonoff DC, Buse JB, Nielsen LL (2008). Exenatide effects on diabetes, obesity, cardiovascular risk factors and hepatic biomarkers in patients with type 2 diabetes treated for at least 3 years. *Current Medical Research and Opinion*.

[31] Jendle J, Nauck MA, Matthews DR (2009). LEAD-2 and LEAD-3 study groups. weight loss with liraglutide, a once-daily human glucagon-like peptide-1 analogue for type 2 diabetes treatment as monotherapy or added to metformin, is primarily as a result of a reduction in fat tissue. *Diabetes, Obesity and Metabolism*.

[32] Drab SR (2010). Incretin-based therapies for type 2 diabetes mellitus: current status and future prospects. *Pharmacotherapy*.

[33] Blonde L, Russell-Jones D (2009). The safety and efficacy of liraglutide with or without oral antidiabetic drug therapy in type 2 diabetes: an overview of the LEAD 1-5 studies. *Diabetes, Obesity and Metabolism*.

[34] Wang W, Walker JR, Wang X (2009). Identification of small-molecule inducers of pancreatic *β*-cell expansion. *Proceedings of the National Academy of Sciences of the United States of America*.

[35] Wajchenberg BL (2007). Beta-cell failure in diabetes and preservation by clinical treatment. *Endocrine Reviews*.

[36] Kendall DM, Riddle MC, Rosenstock J (2005). Effects of exenatide (exendin-4) on glycemic control over 30 weeks in patients with type 2 diabetes treated with metformin and a sulfonylurea. *Diabetes Care*.

[37] Buse JB, Henry RR, Han J, Kim DD, Fineman MS, Baron AD (2004). For the e xenatide-113 clinical study group. effects of exenatide (exendin-4) on glycemic control over 30 weeks in sulfonylurea-treated patients with type 2 diabetes. *Diabetes Care*.

[38] DeFronzo RA, Ratner RE, Han J, Kim DD, Fineman MS, Baron AD (2005). Effects of exenatide (exendin-4) on glycemic control and weight over 30 weeks in metformin-treated patients with type 2. *Diabetes Care*.

[39] Blonde L, Klein EJ, Han J (2006). Interim analysis of the effects of exenatide treatment on A1C, weight and cardiovascular risk factors over 82 weeks in 314 overweight patients with type 2 diabetes. *Diabetes, Obesity and Metabolism*.

[40] Bunck MC, Diamant M, Cornér A (2009). One-year treatment with exenatide improves beta-cell function, compared with insulin glargine, in metformin-treated type 2 diabetic patients: a randomized, controlled trial. *Diabetes Care*.

[41] Heine RJ, Van Gaal LF, Johns D, Mihm MJ, Widel MH, Brodows RG (2005). GWAA study group. exenatide versus insulin glargine in patients with suboptimally controlled type 2 diabetes: a randomized trial. *Annals of Internal Medicine*.

[42] Barnett AH, Burger J, Johns D (2007). Tolerability and efficacy of exenatide and titrated insulin glargine in adult patients with type 2 diabetes previously uncontrolled with metformin or a sulfonylurea: a multinational, randomized, open-label, two-period, crossover noninferiority trial. *Clinical Therapeutics*.

[43] Nauck MA, Duran S, Kim D (2007). A comparison of twice-daily exenatide and biphasic insulin aspart in patients with type 2 diabetes who were suboptimally controlled with sulfonylurea and metformin: a non-inferiority study. *Diabetologia*.

[44] Fakhoury WK, LeReun C, Wright D (2010). A meta-analysis of placebo-controlled clinical trials assessing the efficacy and safety of incretin-based medications in patients with type 2 diabetes. *Pharmacology*.

[45] Derosa G, Maffioli P, Salvadeo SA (2010). Exenatide versus glibenclamide in patients with diabetes. *Diabetes Technology & Therapeutics *.

[46] Marre M, Shaw J, Brändle M  (2009). LEAD-1 SU study group. liraglutide, a once-daily human GLP-1 analogue, added to a sulphonylurea over 26 weeks produces greater improvements in glycaemic and weight control compared with adding rosiglitazone or placebo in subjects with type 2 diabetes (LEAD-1 SU). *Diabetic Medicine*.

[47] Nauck M, Frid A, Hermansen K (2009). LEAD-2 study group. efficacy and safety comparison of liraglutide, glimepiride, and placebo, all in combination with metformin, in type 2 diabetes: the LEAD (liraglutide effect and action in diabetes)-2 study. *Diabetes Care*.

[48] Russell-Jones D, Vaag A, Schmitz O (2009). Liraglutide vs insulin glargine and placebo in combination with metformin and sulfonylurea therapy in type 2 diabetes mellitus (LEAD-5 met+SU): a randomized controlled trial liraglutide effect and action in diabetes 5 (LEAD-5) met+SU study group. *Diabetologia*.

[49] Buse JB, Rosenstock J, Sesti G (2009). LEAD-6 study group.liraglutide once a day versus exenatide twice a day for type 2 diabetes: a 26-week randomised, parallel-group, multinational, open-label trial (LEAD-6). *Lancet*.

[50] Sheffield CA, Kane MP, Busch RS (2008). Safety and efficacy of exenatide in combination with insulin in patients with type 2 diabetes mellitus. *Endocrine Practice*.

[51] Scheen AJ (2010). Pharmacokinetics of dipeptidylpeptidase-4 inhibitors. *Diabetes, Obesity and Metabolism: *.

[52] Scheen AJ (2010). Dipeptidylpeptidase-4 inhibitors (gliptins): focus on drug-drug interactions. *Clin Pharmacokinet *.

[53] Deacon CF (2011). Dipeptidyl peptidase-4 inhibitors in the treatment of type 2 diabetes: a comparative review. *Diabetes, Obesity and Metabolism*.

[54] Foley JE, Sreenan S (2009). Efficacy and safety comparison between the DPP-4 inhibitor vildagliptin and the sulfonylurea gliclazide after two years of monotherapy in drug-naïve patients with type 2 diabetes. *Horm Metab Res*.

[55] D’Alessio DA, Denney AM, Hermiller LM (2009). Treatment with the dipeptidyl peptidase-4 inhibitor vildagliptin improves fasting islet-cell function in subjects with type 2 diabetes. *Journal of Clinical Endocrinology and Metabolism*.

[56] Foley JE, Jordan J (2010). Weight neutrality with the DPP-4 inhibitor, vildagliptin: mechanistic basis and clinical experience. *Vascular Health and Risk Management*.

[57] Ligueros-Saylan M, Foley JE, Schweizer A, Couturier A, Kothny W (2010). An assessment of adverse effects of vildagliptin versus comparators on the liver, the pancreas, the immune system, the skin and in patients with impaired renal function from a large pooled database of phase II and III clinical trials. *Diabetes, Obesity and Metabolism*.

[58] Haidinger M, Werzowa J, Voigt HC (2010). A randomized, placebo-controlled, double-blind, prospective trial to evaluate the effect of vildagliptin in new-onset diabetes mellitus after kidney transplantation. *Trials*.

[59] Banerji MA, Purkayastha D, Francis BH (2010). Safety and tolerability of vildagliptin vs. thiazolidinedione as add-on to metformin in type 2 diabetic patients with and without mild renal impairment: a retrospective analysis of the GALIANT study. *Diabetes Research and Clinical Practice *.

[60] Hanefeld M, Herman GA, Wu M, Mickel C, Sanchez M, Stein PP (2007). Sitagliptin study 014 investigators.once-daily sitagliptin, a dipeptidyl peptidase-4 inhibitor, for the treatment of patients with type 2 diabetes. *Current Medical Research and Opinion*.

[61] Bailey CJ, Green BD, Flatt PR (2010). Fixed-dose combination therapy for type 2 diabetes: sitagliptin plus pioglitazone. *Expert Opinion on Investigational Drugs *.

[62] Engel SS, Williams-Herman DE, Golm GT (2010). Sitagliptin: review of preclinical and clinical data regarding incidence of pancreatitis. *International Journal of Clinical Practice*.

[63] Scheen AJ (2010). Saxagliptin (Onglyza): new inhibitor of the dipeptidylpeptidase-4 for the oral treatment of type 2 diabetes. *Revue Medicale De Liege*.

[64] Rosenstock J, Aguilar-Salinas C, Klein E (2009). Effect of saxagliptin monotherapy in treatment-naïve patients with type 2 diabetes. *Current Medical Research and Opinion*.

[65] Jadzinsky M, Pfützner A, Paz-Pacheco E (2009). Saxagliptin given in combination with metformin as initial therapy improves glycaemic control in patients with type 2 diabetes compared with either monotherapy: a randomized controlled trial. *Diabetes, Obesity and Metabolism*.

[66] Chacra AR, Tan GH, Apanovitch A, Ravichandran S, List J, Chen R (2009). Saxagliptin added to a submaximal dose of sulphonylurea improves glycaemic control compared with uptitration of sulphonylurea in patients with type 2 diabetes: a randomised controlled trial. *International Journal of Clinical Practice*.

[67] Hollander P, Li J, Allen E, Chen R (2009). For the CV181-013 investigators.saxagliptin added to a thiazolidinedione improves glycemic control in patients with type 2 diabetes and inadequate control on thiazolidinedione alone. *Journal of Clinical Endocrinology and Metabolism*.

[68] DeFronzo RA, Hissa M, Garber AJ (2009). The efficacy and safety of saxagliptin when added to metformin therapy in patients with inadequately controlled type 2 diabetes with metformin alone. *Diabetes Care*.

[69] Nowicki M, Rychlik I, Haller H, Warren ML, Suchower L, Gause-Nilsson I (2011). Saxagliptin improves glycaemic control and is well tolerated in patients with type 2 diabetes mellitus and renal impairment. *Diabetes, Obesity and Metabolism*.

[70] Scheen AJ, Charpentier G, Ostgren CJ, Hellqvist A, Gause-Nilsson I (2010). Efficacy and safety of saxagliptin in combination with metformin compared with sitagliptin in combination with metformin in adult patients with type 2 diabetes mellitus. *Diabetes Metab Res Rev*.

[71] Bergenstal RM, Wysham C, Macconell L (2010). DURATION-2 study group. efficacy and safety of exenatide once weekly versus sitagliptin or pioglitazone as an adjunct to metformin for treatment of type 2 diabetes (DURATION-2): a randomised trial. *Lancet*.

[72] Mannucci E, Rotella CM (2008). Future perspectives on glucagon-like peptide-1, diabetes and cardiovascular risk. *Nutrition, Metabolism and Cardiovascular Diseases*.

[73] Astrup A, Rössner S, Van Gaal L (2009). NN8022-1807 study group. effects of liraglutide in the treatment of obesity: a randomised, double-blind, placebo-controlled study.. *Lancet*.

[74] Bock G, Dalla Man C, Micheletto F (2010). The effect of DPP-4 inhibition with sitagliptin on incretin secretion and on fasting and postprandial glucose turnover in subjects with impaired fasting glucose. *Clinical Endocrinology (Oxf)*.

[75] Madsbad S, Kielgast U, Asmar M, Deacon C, Torekov SS, Holst JJ (2011). An overview of once-weekly GLP-1 receptor agonists- available efficacy and safety data and perspectives for the future. *Diabetes, Obesity and Metabolism*.

